# Potent CD4^+^ T cell-associated antitumor memory responses induced by trifunctional bispecific antibodies in combination with immune checkpoint inhibition

**DOI:** 10.18632/oncotarget.13888

**Published:** 2016-12-10

**Authors:** Nina Deppisch, Peter Ruf, Nina Eißler, Horst Lindhofer, Ralph Mocikat

**Affiliations:** ^1^ Institut für Molekulare Immunologie, Helmholtz-Zentrum München, Germany; ^2^ Trion Research GmbH, Martinsried, Germany; ^3^ AG Translationale Molekulare Immunologie, Helmholtz-Zentrum München, Germany

**Keywords:** tumor antigen, T-cell activation, melanoma, CTLA-4, cancer immunotherapy

## Abstract

Combinatorial approaches of immunotherapy hold great promise for the treatment of malignant disease. Here, we examined the potential of combining an immune checkpoint inhibitor and trifunctional bispecific antibodies (trAbs) in a preclinical melanoma mouse model using surrogate antibodies of Ipilimumab and Catumaxomab, both of which have already been approved for clinical use. The specific binding arms of trAbs redirect T cells to tumor cells and trigger direct cytotoxicity, while the Fc region activates accessory cells eventually giving rise to a long-lasting immunologic memory. We show here that T cells redirected to tumor cells by trAbs strongly upregulate CTLA-4 expression *in vitro* and *in vivo*. This suggested that blocking of CTLA-4 in combination with trAb treatment enhances T-cell activation in a tumor-selective manner. However, when mice were challenged with melanoma cells and subsequently treated with antibodies, there was only a moderate beneficial effect of the combinatorial approach *in vivo* with regard to direct tumor destruction in comparison to trAb therapy alone. By contrast, a significantly improved vaccination effect was obtained by CTLA-4 blocking during trAb-dependent immunization. This resulted in enhanced rejection of melanoma cells given after pre-immunization. The improved immunologic memory induced by the combinatorial approach correlated with an increased humoral antitumor response as measured in the sera and an expansion of CD4^+^ memory T cells found in the spleens.

## INTRODUCTION

In recent years, the broadened understanding of the interplay between components of the immune system and malignant cells has paved the way for establishing powerful tools of immunotherapy in the clinics. One major goal of cancer immunotherapy is to stimulate tumor-reactive T cells, which are often silenced in the tumor microenvironment. Molecules related to tumor escape from T-cell attack include cytotoxic T lymphocyte-associated protein-4 (CTLA-4) and programmed death-1 (PD-1), which are upregulated on T cells as a counter-regulatory mechanism upon prolonged stimulation [1]. The interaction of these “immune checkpoint” molecules with their ligands, B7.1/B7.2 and PD-L1/PD-L2 expressed on antigen-presenting cells and tumor cells, respectively, inhibits positive signals mediated by the T-cell receptor (TCR) or the costimulatory receptor CD28 and thereby leads to suppression of T-cell responses [2–5]. Therefore, antibody-mediated blocking of immune checkpoints is an effective approach to boost tumor-reactive T-cell functions.

Ipilimumab, Nivolumab and Pembrolizumab are human or humanized monoclonal antibodies (mAb) that target CTLA-4 or PD-1, respectively, and interfere with inhibitory signals delivered by these receptors to the T cell. The CTLA-4-directed mAb Ipilimumab has been approved as first- and second-line therapy for patients with malignant melanoma and showed promising results in terms of overall survival [6, review in 7]. Combination therapies including Ipilimumab and anti-PD-1 [8–10] or other mAbs [11] even proved to be superior to treatment with a single mAb.

A drawback of combining different immune checkpoint inhibitors is their unspecific mode of action involving “off-site” activation of T cells, which gives rise to undesired side effects [12]. Therefore, we established a novel combination therapy making use of only one immune checkpoint inhibitor. This approach allows the activity of Ipilimumab to be targeted to T cells that strongly express CTLA-4 as a consequence of their specific stimulation in the presence of tumor cells. The tumor-specific T-cell activation is secured by trifunctional bispecific antibodies (trAbs), which selectively redirect T cells to tumor cells by virtue of two different binding arms recognizing CD3 and a tumor-associated antigen (TAA), respectively. Additionally, the intact Fc region of trAbs recruits and stimulates accessory cells such as dendritic cells (DCs) or macrophages *via* activating Fc receptors [13, 14]. These cells provide additional stimuli to T cells, take up tumor cell debris and present tumor-derived peptides to the immune system [15, 16]. Thus, trAbs not only lead to T cell-dependent tumor destruction, but also induce a long-lasting tumor-specific immunologic memory [16–18]. The role of the intact Fc region was established by experiments using Fc blocking or Fc-devoid antibody constructs [15–17, 19]. TrAbs are already in clinical use. Catumaxomab, for example, which binds to the TAA epithelial cell adhesion molecule (EpCAM), has been approved for the treatment of malignant ascites [20]. Other trAb constructs are investigated in clinical studies.

In an attempt to endow mAb-mediated blockade of CTLA-4 with increased specificity for tumor-reactive T cells, we examined whether trAb-induced T-cell activation and neutralization of the concomitant CTLA-4 upregulation on T cells cooperate with regard to enhanced tumor rejection and induction of an immunologic memory. A model tumor used in this paper is the B16F0-derived melanoma B78-D14, which is engineered to express GD2 [21]. This ganglioside is a promising antigen for targeting small cell lung cancer and malignancies of neuroectodermal origin such as neuroblastoma, glioma, sarcoma or melanoma in humans [22–24]. We also included the more immunogenic melanoma B16-EpCAM [16], which expresses the antigen recognized by the clinically relevant trAb Catumaxomab [20]. The constructs Surek [17, 19, 25, 26] and BiLu [16] served as surrogate trAbs cross-linking GD2 or EpCAM, respectively, with the CD3 receptor on murine T cells.

## RESULTS

### CTLA-4 is upregulated following trAb-induced T-cell activation

**Figure 1:**
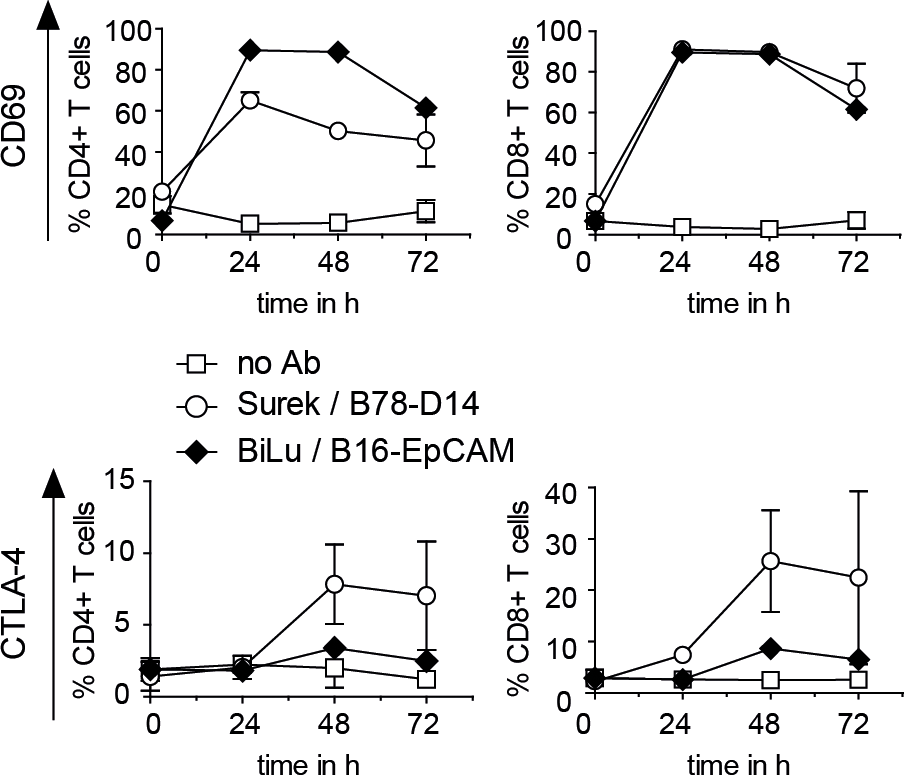
CD69 and CTLA-4 induction on T cells activated with trAbs *in vitro* T cells were cultivated with DCs, irradiated B78-D14 or B16-EpCAM cells and trAb Surek or BiLu, respectively, as outlined in Materials and Methods. At different time points, surface expression of CD69 and CTLA-4 on CD4^+^ and CD8^+^ T cells was determined by FACS analyses. Mean values and standard deviations from 3 independent experiments are shown.

It was anticipated that the strong CD3-mediated T-cell activation induced by tumor-directed trAbs not only ignites T-cell effector functions, but also entails CTLA-4 upregulation on the surface of activated T cells. For combining anti-CTLA-4 treatment with trAb therapy, it is necessary to establish the upregulation of CTLA-4 following trAb-dependent activation. Therefore, we determined CD69 and CTLA-4 levels at different time points after *in vitro* incubation of T cells isolated from mouse spleens together with DCs and tumor cells (B78-D14 or B16-EpCAM) in the presence of Surek or BiLu. While the T-cell activation marker CD69 already increased by day 1, CTLA-4 expression only peaked after 48 to 72 hours (Figure 1).

**Figure 2:**
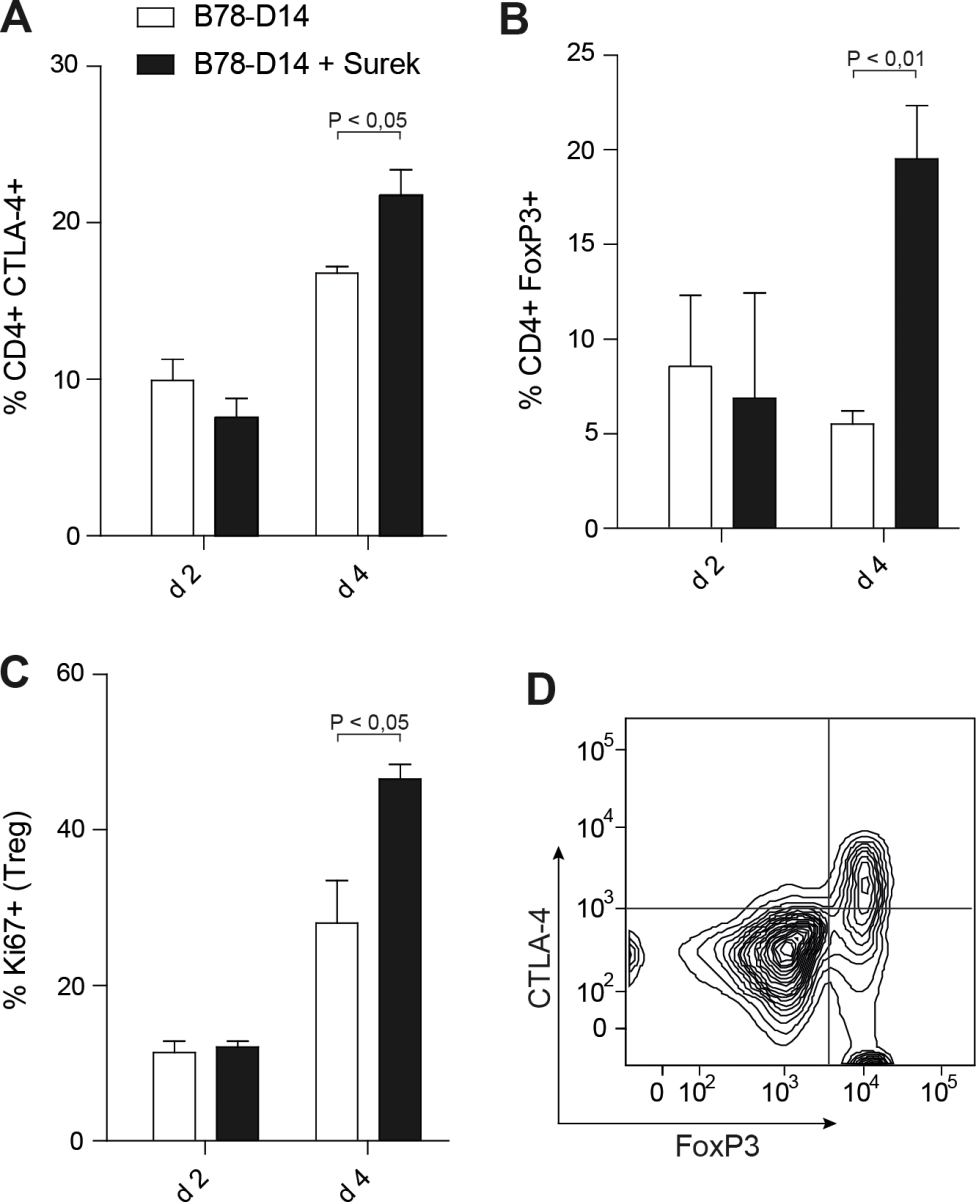
Counter-regulatory mechanisms induced by trAb treatment *in vivo* Mice received irradiated B78-D14 cells with or without 10 μg of trAb Surek i.p. After 2 and 4 days, spleens were isolated and CD4^+^ T cells were phenotypically characterized. (**A**) Expression of CTLA-4 on CD4^+^ T cells. (**B**) Percentages of CD4^+^FoxP3^+^ cells. (**C**) Ki67 as a proliferation marker was stained in CD4^+^FoxP3^+^ T cells. (**D**) CTLA-4 upregulation is mainly restricted to the Treg population. Gating was done for CD4^+^ cells. At least 3 animals were included in each group. Columns indicate means and SEM. Statistics was done using the Mann-Whitney test.

To examine CTLA-4 expression *in vivo*, a single dose of Surek was injected into mice along with irradiated B78-D14 cells. In this setting, a significant upregulation of CTLA-4 was observed after 4 days (Figure 2A). The frequency of FoxP3^+^ CD4^+^ regulatory T cells (Tregs) was also markedly increased at this time point (Figure 2B). As shown by Ki67 staining, this was likely due to enhanced Treg proliferation (Figure 2C). Interestingly, CTLA-4 upregulation on the CD4^+^ T cells was mainly restricted to the FoxP3^+^ population (Figure 2D). Since CTLA-4 mediates suppressive functions of Tregs, the data supported the concept of blocking this molecule during trAb therapy.

### Direct tumor killing is moderately improved by combining trAb and anti-CTLA-4 treatment

**Figure 3:**
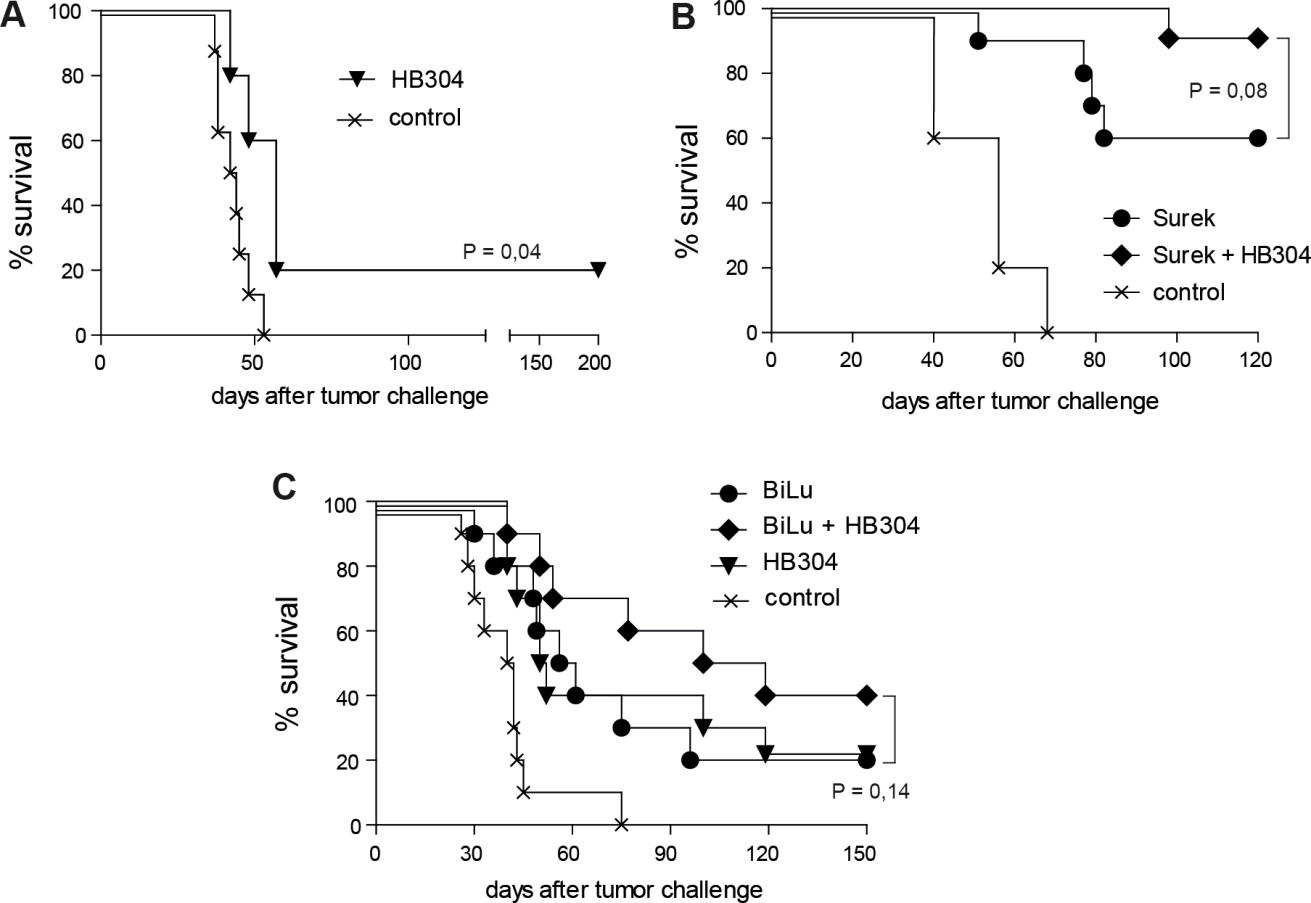
Direct trAb-mediated tumor destruction is moderately improved by combining trAb and anti-CTLA-4 therapy Antibody treatment of mice started 2 days after challenge with 10^5^ B78-D14 or B16-EpCAM cells. In the experiments shown, 5 to 10 mice were included. (**A**) Blocking of CTLA-4 alone by HB304 has only a marginal effect on tumor killing. (**B**) Survival of mice after therapy with Surek alone or with Surek simultaneously delivered with HB304. (**C**) Moderate survival benefit of mice treated with trAb BiLu and HB304 in comparison to monotherapy in the B16-EpCAM model. Significances were determined using the logrank test.

We then evaluated the therapeutic potential of a combined trAb/anti-CTLA-4 treatment *in vivo* in comparison to monotherapy. Based on previous experiments [25], the tumor models were adjusted to suboptimal antibody doses to secure detection of any synergisms of the combination approach. Therapy started 2 days after a lethal challenge with B78-D14 melanoma. Treatment with the anti-CTLA-4 mAb HB304 alone had only a marginal effect (Figure 3A), while monotherapy with Surek rescued up to 60% of mice bearing an established B78-D14 burden (Figure 3B). When both antibodies were combined, however, the overall survival of mice increased to 90% (Figure 3B). The data indicate that the approach combining both antibodies has a beneficial effect as compared to Surek monotherapy albeit with a significance of P = 0.08 (logrank).

These results could be verified in the B16-EpCAM model, although the absolute survival rates cannot be compared in the two models. Therapy with either the trAb BiLu or the anti-CTLA-4 mAb HB304 alone yielded identical survival benefits in comparison to the tumor control group. This effect could be improved by combining both therapy regimens resulting in a prolongation of the median survival and an increase of long-term survivors from 20 to 40% (Figure 3C).

### Tumor vaccination by trAb Surek is strongly boosted by blockade of CTLA-4

**Figure 4:**
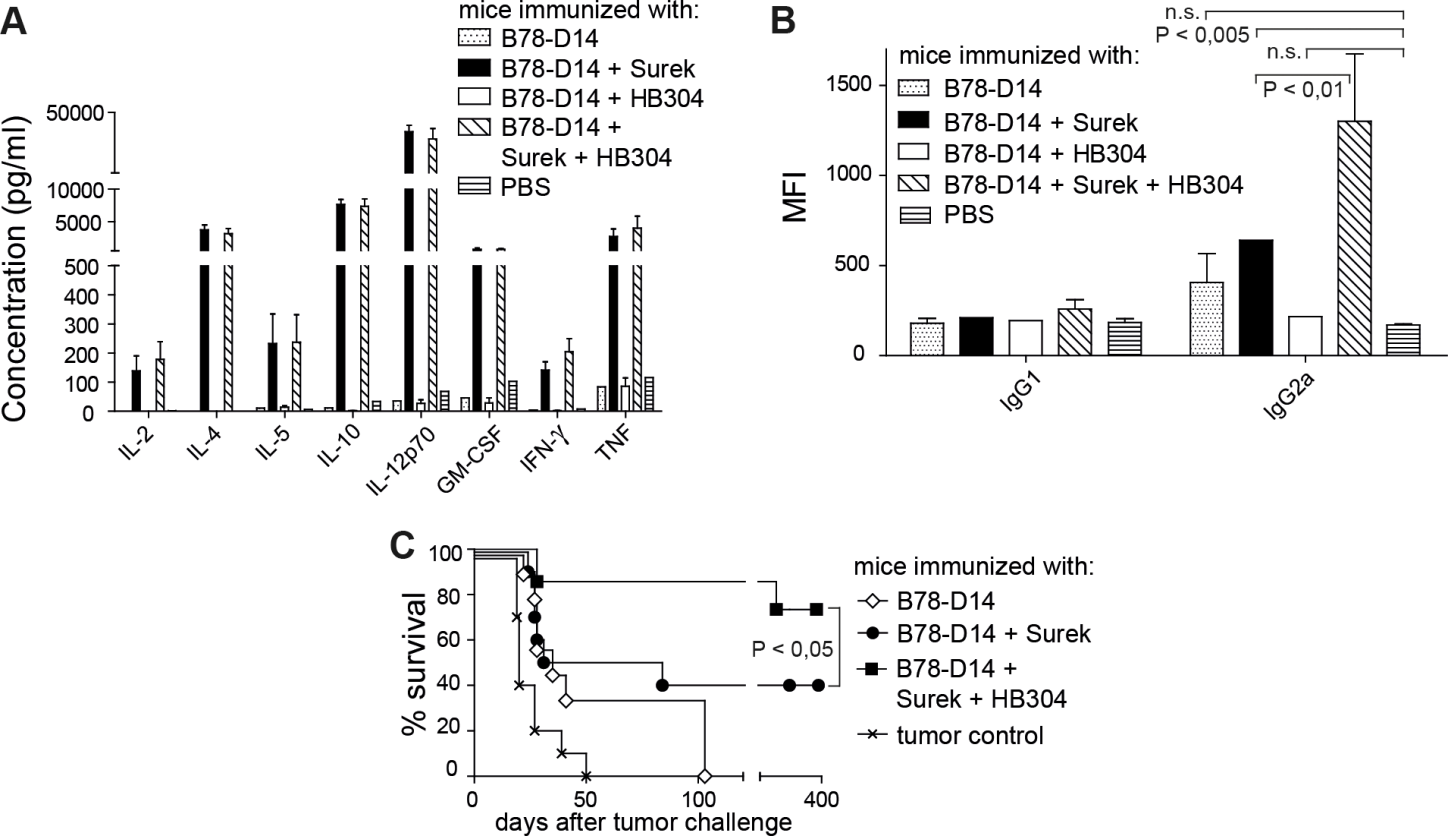
Immunologic memory induced by trAb Surek with or without CTLA-4 blocking Immunization was done as outlined in Materials and Methods using the indicated immunogens. Irradiated B78-D14 cells as an antigen source were included in each setting. (**A**) Th1/Th2 cytokines in sera of mice on day 21 after starting immunization. Up to 8 mice were included in each group. For all cytokines tested, the difference between the Surek and the Surek/HB304 group was not significant, while these two groups were different from all other groups with *P <* 0.005 (Mann-Whitney). (**B**) Tumor-directed antibodies in sera of mice vaccinated with different immunogens. Binding of serum IgG1 and IgG2a antibodies to melanoma cells was measured by flow cytometry. Mean fluorescence intensities (MFI) are shown as a quantitative measure for antibody coating of tumor cells. Up to 5 animals were included in each group. The columns show means and SEM. Significances (Mann-Whitney) are indicated. The differences between IgG1 titers are not significant. (**C**) On day 21, mice were challenged with a lethal dose of melanoma cells. The significance is denoted (logrank).

Taken together, a moderate bonus effect on direct tumor killing may be achieved by using the combination approach. Since trAbs not only mediate direct tumor cell killing, but also elicit a vaccination effect [17], we then asked whether the immunologic memory induced by trAbs is affected by the combination with anti-CTLA-4 treatment. These experiments were only done using the B78-D14 melanoma because the B16-EpCAM model is less stringent due to its higher inherent immunogenicity (our unpublished data). Since effective long-lasting tumor protection is dependent on T-cell responses of the Th1 type [27, 28], we first compared the levels of Th1 and Th2 cytokines in sera of mice that had been vaccinated with trAb Surek or HB304 or the antibody combination together with replication-incompetent tumor cells as an antigen source. After Surek immunization, high concentrations of Th1 cytokines were detected (Figure 4A). The data confirmed our previous results, which indicated a clear Th1 bias involving IL-12 expression by DCs and IFN-γ production by T cells [17]. Accordingly, an upregulation of Th2 cytokines was also observed, because this is a prerequisite for inducing Th1 responses (see Discussion). In contrast, HB304 alone did not induce any cytokine response. Furthermore, the cytokine levels that were induced by Surek could not be further enhanced by combining trAb treatment with HB304 (Figure 4A). Likewise, when T cells from vaccinated mice were restimulated with melanoma-derived peptides *in vitro*, the combination group neither showed enhanced IFN-γ release as compared to animals vaccinated with Surek alone (not shown).

Since it is established that trAb-dependent vaccination induces tumor-specific humoral immune responses [16], which correlate with tumor protection *in vivo*, we then measured the titers of melanoma-directed antibodies in sera of vaccinated mice. While specific antibodies of the IgG1 subclass were not significantly affected by any vaccination protocol, immunization with Surek induced elevated levels of the IgG2a subclass (Figure 4B). HB304 induced anti-melanoma antibody titers that were not significantly different from untreated controls. However, combining Surek with HB304 strongly enhanced the IgG2a induction effected by Surek monotherapy (Figure 4B).

As the humoral IgG2a response is a predictive parameter for the immunologic antitumor memory [16], we then compared Surek and Surek/HB304 vaccination with regard to melanoma rejection *in vivo*. Challenge with a lethal dose of viable melanoma cells three weeks after start of the immunization showed that the vaccination effect induced by Surek is indeed significantly enhanced when the trAb is combined with HB304 during the immunization phase. While 40% of mice survived in the Surek-immunized group, about 80% were effectively protected when both antibodies were used for vaccination (Figure 4C). Thus, the bonus effect of the combination approach appeared far more pronounced in terms of anti-tumor memory than direct tumor cell killing.

### The combination of trAb Surek and anti-CTLA-4 induces CD4^+^ memory cells

**Figure 5:**
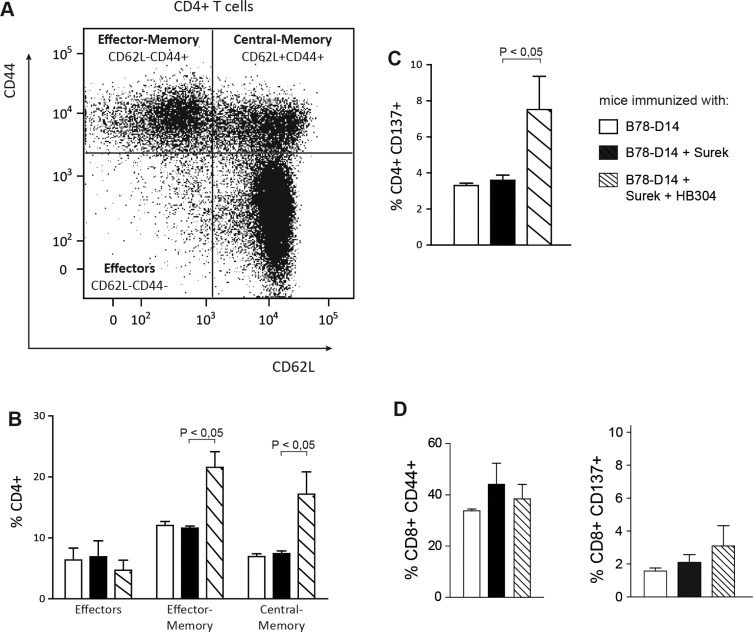
Characterization of memory T cells after immunization with Surek alone or in combination with CTLA-4 blocking T cells from spleens of differentially vaccinated mice were stimulated *in vitro* with splenocytes that were loaded with melanoma-derived peptides [17]. After 7 days, T cells were analyzed for expression of CD4, CD8, CD44 and CD62L. At least 4 mice were included in each group. (**A**) Representative diagram delineating the differentiation between different memory and effector T-cell compartments. (**B**) Quantitation of CD4^+^ T-cell subpopulations from differentially vaccinated mice. (**C**) Expression of CD137 on CD4^+^ T cells after *in vivo* immunization and restimulation with melanoma peptides. CD137^+^ cells were exclusively identified as CD44^+^ memory cells (not shown). (**D**) In the CD8^+^ T-cell population, an expansion of the memory compartment or a significant upregulation of CD137 was not induced by the combination approach. In all panels, columns represent mean values and SEM. Significances are also shown (Mann-Whitney).

Having shown that the combinatorial vaccination improves the humoral IgG2a antitumor response and the long-lasting protection against melanoma as compared to trAb immunization alone, we analyzed in more detail the T-cell compartment in differentially immunized mice. To discriminate effector, effector-memory and central-memory cells, T cells isolated from immunized mice and restimulated *in vitro* were analyzed for expression of CD44 and CD62L (Figure 5A). It turned out that the fraction of memory CD4^+^ T cells was markedly increased in the group having undergone the combinatorial vaccination (Figure 5B).

CD137 is a costimulatory molecule that is expressed on the surface of T cells upon antigen-specific and TCR-dependent stimulation. Interestingly, CD137 was elevated on CD4^+^ T cells that had been primed *in vivo* by irradiated tumor cells and Surek together with HB304 (Figure 5C). These CD137^+^ cells were exclusively identified as CD44^+^ memory cells (data not shown). By contrast, neither an expansion of the memory compartment nor a significant upregulation of CD137 was seen in the CD8^+^ T-cell population (Figure 5D).

## DISCUSSION

Combinatorial approaches of cancer immunotherapy have attracted much interest in the past years. Specifically, the simultaneous blockade of different immune checkpoints has been extensively evaluated in translational and clinical studies [8–10]. The rationale is to release the brake imposed to *bona fide* tumor-reactive T cells by surface molecules like CTLA-4, whose induction is considered as a counter-regulatory mechanism required for self-limitation of any immune response [29, 30]. However, tumor escape may be more effectively counteracted if reagents with different modes of action are combined. Therefore, we nourished the blockade of CTLA-4 with trAbs that specifically redirect T cells to TAAs and activate the T cells in the presence of tumor cells. Thus, the unspecific effect of anti-CTLA-4 is supplemented with an antigen-specific component because after trAb treatment, specifically activated T cells with induced CTLA-4 expression can be further stimulated by anti-CTLA-4 mAbs. As a consequence, the redirection of activated T cells by trAbs could be beneficial for the side-effect profile of checkpoint inhibitors. This is indicated by experiments using an allogeneic transplantation setting, which also inherently bears the danger of tissue damage by uncontrolled T-cell activity. Here, T cells could be redirected away from healthy tissues to the tumor site by trAbs, thereby significantly reducing graft-versus-host-disease [31].

In other studies, the antitumor efficacy of bispecific antibody constructs cross-linking HER2 or CD33 with CD3 could also be improved by blocking PD-L1, which is expressed on tumor cells and inhibits T-cell responses *via* ligation of PD-1 [32, 33]. In contrast to these constructs, trAbs have a unique Fc region comprising the mouse IgG2a and the rat IgG2b isotype, which enables the preferential interaction with activating Fc receptors [14]. Thus, trAbs not only mediate direct cell lysis but also induce a long-lasting T cell-dependent immunologic antitumor memory, because accessory cells expressing activating Fc receptors are stimulated and present tumor-derived peptides to the immune system [13, 15–17]. The vaccination effect exerted by trAbs involves a polyvalent T-cell response, which is directed against numerous TAAs and does not depend on the antigen targeted by the therapeutic trAb [16, 17]. This was shown by challenging mice with the parental melanoma cell line B16F0, which did not express the antigens recognized by the trAb used for vaccination. Polyvalent immune responses are thought to be superior to immunity against single TAAs because tumor immune escape due to antigen loss becomes less likely [34, 35].

A major aim of our study was therefore to examine the trAb-induced vaccination effect in combination with CTLA-4 blockade. Importantly, while direct trAb-mediated destruction of tumor cells was only moderately improved by combination with CTLA-4 inhibition, the immunologic memory elicited by the combination treatment was indeed considerably enhanced. The memory induced was associated with an expansion of CD4^+^ memory cells, whose CD137 expression indicated preceding antigen-specific activation (Figure 5). These phenotypic changes were not seen in the CD8^+^ T-cell compartment.

Because a similar increase of Th1 cytokines was seen in all trAb-vaccinated groups (Figure 4A), the combination approach had no impact on the Th1 response, which is instrumental for effective tumor protection [27, 28]. The simultaneous upregulation of Th2 cytokines in all groups where Th1 cytokines were induced was not unexpected since Th2 cytokines are required for priming Th1 responses [34, 36, 37]. A clear hierarchy, however, was detected for the humoral immune response induced by the different antibodies (Figure 4B). Thus, the improved immunologic memory observed after vaccination with trAbs combined with CTLA-4 blockade correlated with increased serum titers of melanoma-reactive IgG2a antibodies. Indeed, humoral immunity has been shown to be relevant for tumor protection *in vivo* [16]. The finding of CD4^+^ rather than CD8^+^ memory cells after combined vaccination (Figure 5) supports this concept.

It is well known that CTLA-4 blocking increases Treg proliferation [38]. Accordingly, the proliferation and the amounts of Tregs were enhanced after combinatorial vaccination. Importantly, this increased Treg proliferation was not responsible for the expansion of the antigen-experienced memory CD4^+^ T cells (manuscript in preparation), as might be predicted by a study using another tumor model where Tregs primarily contributed to the CD137^+^CD44^+^CD4^+^ memory compartment [39].

Thus, the improved immunization effect did not depend on a reduction of Treg numbers, but possibly (at least partly) on a functional inhibition of Tregs. As it has been shown that Tregs control B-cell expression of CD80 and CD86 *via* the coreceptor CTLA-4 [40], a potential mechanism enhancing the vaccination effect of trAbs in the combination setting may be mediated by CTLA-4 blockade on Tregs, thus fostering the induction of antitumor antibodies (Figure 4B). Further mechanistic studies, which should also address the role of follicular helper cells, are warranted.

Up to now, checkpoint inhibitors have been most effective in melanoma, bladder cancer and lung cancer. Other types of malignant disease where checkpoint inhibitors still have to prove their effectiveness should also be addressed by combining with trAb immunization. Therefore, the positive effect of blocking CTLA-4 during trAb vaccination on the CD4^+^ T cell-associated antitumor memory argues for further evaluation of this concept in preclinical and clinical settings.

## MATERIALS AND METHODS

### Generation of antibodies

TrAbs were produced by quadroma technology as described [16, 25]. Quadroma-derived supernatants were purified by protein A chromatography applying sequential pH elution followed by a cationic exchange chromatography purification step. Surek [17, 19, 25, 26] is a trAb that was generated by fusion of the parental hybridomas 17A2 (anti-mouse CD3, rat IgG2b) and Me361 (anti-GD2, mouse IgG2a) [24]. BiLu [16] comprises the same anti-CD3 specificity and additionally includes a mouse IgG2a binding arm recognizing human EpCAM, which was derived from the clone C215 [16]. For production of HB304 (anti-mouse CTLA-4), the hamster hybridoma clone UC10-4F10-11 [2] was used. Cell lines were cultivated in chemically defined protein-free medium. All antibodies were manufactured by Trion Research GmbH.

### Cultivation of B78-D14 and B16-EpCAM tumor cells

B78-D14 [21] and B16-EpCAM [16] cells were grown in RPMI 1640 medium supplemented with 8.9% and 5%, respectively, fetal calf serum, 2 mM L-glutamine, 1 mM sodium pyruvate, and 1× nonessential amino acids. Further, 400 μg/ml G418 and 500 μg/ml Hygromycin B were added to B78-D14. Cells were extensively washed in PBS before application. The identity of the cell lines was regularly confirmed on the basis of cell morphology and the expression of the transgenes.

### Tumor protection and vaccination experiments

C57BL/6 mice were purchased from Taconic (Ry, Denmark). All animal experiments were performed at least twice with 5 to 10 female animals included in each group. For testing trAb-induced tumor rejection, mice received a challenge of 10^5^ B78-D14 or B16-EpCAM cells and were treated on day 2 and 5 with 50 μg Surek or 10 μg BiLu. 100 μg HB304 were either given simultaneously with trAbs or, in some experiments, repeatedly on days 9 and 12 followed by four other injections in weekly intervals.

To examine the long-term immunologic memory, mice were immunized on day 0 and 14 with 10^5^ irradiated B78-D14 cells (100 Gy) together with 10 μg Surek. Other groups were additionally treated with 100 μg HB304 on days 4, 8, 13 and 20. On day 21, all mice received a challenge of 3 × 10^3^ viable B16F0 melanoma cells.

All cells and antibodies were delivered i.p. except for B16-EpCAM cells that were i.v. administered. Control groups receiving tumor cells and PBS only were included in each experiment. Mice were euthanized when signs of tumor growth became visible. All animal experiments were in accordance with animal welfare regulations and had been approved by the competent authority.

### Phenotyping of T cells

T-cell analyses were performed by fluorescence-activated cell sorting (FACS) using an LSRII or a FACS Calibur flow cytometer and the cell quest analysis program (BD Bioscience, Heidelberg, Germany). T-cell surface markers were directly stained with fluorescence-labeled mAbs against CD4 (clone RM4-5; BD Biosciences), CD8 (53-6.7, eBioscience, San Diego, USA), CD44 (IM7; BD Biosciences), CD62L (MEL-14; eBioscience) and CD69 (H1.2F3; BD Bioscience). Ki67 (SolA15; eBioscience), CTLA-4 (UC10-4B9; BioLegend, or HB304) and the transcription factor FoxP3 (FJK-16s; eBioscience) were stained intracellularly.

### TrAb-mediated T-cell stimulation *in vitro*

Immature DCs were prepared by culturing bone marrow precursors from C57BL/6 mice in RPMI 1640 supplemented with 20% FCS, 2 mM L-glutamine, 100 U/ml penicillin and streptomycin, 50 μM 2-mercaptoethanol, sodium pyruvate and nonessential amino acids in the presence of 100 ng/ml granulocyte-macrophage colony-stimulating factor (GM-CSF). Medium was replaced every second day, cells were frozen at -140°C on day 7. Frozen DCs were thawed, counted and directly applied to T-cell stimulation assays.

4 × 10^6^ T-cells, which were isolated from spleens of naïve C57BL/6 mice by panning of B lymphocytes with anti-IgG+M antibodies (Dianova, Hamburg, Germany), were co-cultivated with 2 × 10^5^ DCs and 10^5^ irradiated (100 Gy) tumor cells in 24-well plates for three days. TrAbs were added at 1 μg/ml. Cells were cultivated in RPMI 1640 medium supplemented with 10% fetal calf serum, 2 mM L-Glutamine, 1 mM sodium pyruvate, 1× non-essential amino acids, 10 mM HEPES and 50 μM 2-mercaptoethanol. CD69 and CTLA-4 surface expression of CD4^+^ and CD8^+^ T cells was determined by flow cytometry at 0, 24, 48 and 72 h using the mAbs listed above. The percentage of positive cells was determined in comparison to corresponding isotype controls.

### *In-vitro* stimulation of T cells using peptide-loaded splenocytes

*In-vitro* stimulation of T cells was conducted by co-culturing 5 × 10^6^ splenocytes of immunized mice with 5 × 10^6^ splenocytes of naïve mice, which were loaded with Trp-2, MAGE-A5 and gp100 peptides [17] and irradiated at 30 Gy, in the presence of IL-2 (30 U/ml; Amersham Pharmacia, Freiburg, Germany) in 24-well plates. After 7 days, T cells were harvested and characterized by FACS analysis as described above.

To determine IFN-γ release of activated T cells, 5 × 10^5^ splenocytes of immunized mice were co-cultured with 5 × 10^5^ peptide-loaded splenocytes of naïve mice in 96-well plates. Supernatants were collected 24 h and 48 h later to measure IFN-γ concentrations.

### Cytokine quantitation

IFN-γ in supernatants was quantitated by enzyme-linked immunosorbent assay (Ready-SET-go IFN-γ ELISA Kit; eBioscience) according to the manufacturer´s instructions. Th1/Th2 cytokines in sera of mice were analyzed by using the Bio-Plex^TM^ cytokine assay (Bio-Rad, München, Germany).

### Analysis of tumor-reactive antibodies

Tumor-specific antibodies were analyzed in sera after immunization and tumor challenge as described above. Sera were taken about 3 weeks following the last trAb injection. At this time point, no residual trAb was detected. Tumor cells were pre-incubated with sera on ice; after washing cells, fluorescence-labeled antibody against mouse IgG1 or IgG2a was added. Analysis of IgG-binding tumor cells was subsequently performed using FACS (LSRII flow cytometer; BD Biosciences).

## References

[1] Wherry EJ (2011). T cell exhaustion. Nat Immunol.

[2] Walunas TL, Lenschow DJ, Bakker CY, Linsley PS, Freeman GJ, Green JM, Thompson CB, Bluestone JA (1994). CTLA-4 can function as a negative regulator of T cell activation. Immunity.

[3] Freeman GJ, Long AJ, Iwai Y, Bourque K, Chernova T, Nishimura H, Fitz LJ, Malenkovich N, Okazaki T, Byrne MC, Horton HF, Fouser L, Carter L (2000). Engagement of the PD-1 immunoinhibitory receptor by a novel B7 family member leads to negative regulation of lymphocyte activation. The J Exp Med.

[4] Collins AV, Brodie DW, Gilbert RJ, Iaboni A, Manso-Sancho R, Walse B, Stuart DI, van der Merwe PA, Davis SJ (2002). The interaction properties of costimulatory molecules revisited. Immunity.

[5] Parry RV, Chemnitz JM, Frauwirth KA, Lanfranco AR, Braunstein I, Kobayashi SV, Linsley PS, Thompson CB, Riley JL (2005). CTLA-4 and PD-1 receptors inhibit T-cell activation by distinct mechanisms. Mol Cell Biol.

[6] Hodi FS, O’Day SJ, McDermott DF, Weber RW, Sosman JA, Haanen JB, Gonzalez R, Robert C, Schadendorf D, Hassel JC, Akerley W, van den Eertwegh AJ, Lutzky J (2010). Improved survival with ipilimumab in patients with metastatic melanoma. N Engl J Med.

[7] Blank CU, Enk A (2014). Therapeutic use of anti-CTLA-4 antibodies. Int Immunol.

[8] Larkin J, Chiarion-Sileni V, Gonzalez R, Grob JJ, Cowey CL, Lao CD, Schadendorf D, Dummer R, Smylie M, Rutkowski P, Ferrucci PF, Hill A, Wagstaff J (2015). Combined nivolumab and ipilimumab or monotherapy in untreated melanoma. N Engl J Med.

[9] Postow MA, Chesney J, Pavlick AC, Robert C, Grossmann K, McDermott D, Linette GP, Meyer N, Giguere JK, Agarwala SS, Shaheen M, Ernstoff MS, Minor D (2015). Nivolumab and ipilimumab versus ipilimumab in untreated melanoma. N Engl J Med.

[10] Wolchok JD, Kluger H, Callahan MK, Postow MA, Rizvi NA, Lesokhin AM, Segal NH, Ariyan CE, Gordon RA, Reed K, Burke MM, Caldwell A, Kronenberg SA (2013). Nivolumab plus ipilimumab in advanced melanoma. N Engl J Med.

[11] Dai M, Yip YY, Hellstrom I, Hellstrom KE (2015). Curing mice with large tumors by locally delivering combinations of immunomodulatory antibodies. Clin Cancer Res.

[12] Tsai KK, Daud AI (2015). Nivolumab plus Ipilimumab in the treatment of advanced melanoma. J Hematol Oncol.

[13] Zeidler R, Reisbach G, Wollenberg B, Lang S, Chaubal S, Schmitt B, Lindhofer H (1999). Simultaneous activation of T cells and accessory cells by a new class of intact bispecific antibody results in efficient tumor cell killing. J Immunol.

[14] Lindhofer H, Hess J, Ruf P (2011). Trifunctional Triomab® antibodies for cancer therapy.. In: Kontermann RE (ed.), Bispecific antibodies.

[15] Zeidler R, Mysliwietz J, Csanady M, Walz A, Ziegler I, Schmitt B, Wollenberg B, Lindhofer H (2000). The Fc-region of a new class of intact bispecific antibody mediates activation of accessory cells and NK cells and induces direct phagocytosis of tumour cells. Br J Cancer.

[16] Ruf P, Lindhofer H (2001). Induction of a long-lasting antitumor immunity by a trifunctional bispecific antibody. Blood.

[17] Eißler N, Ruf P, Mysliwietz J, Lindhofer H, Mocikat R (2012). Trifunctional bispecific antibodies induce tumor-specific T cells and elicit a vaccination effect. Cancer Res.

[18] Atanackovic D, Reinhard H, Meyer S, Spöck S, Grob T, Luetkens T, Yousef S, Cao Y, Hildebrandt Y, Templin J, Bartels K, Lajmi N, Stoiber H (2013). The trifunctional antibody catumaxomab amplifies and shapes tumor-specific immunity when applied to gastric cancer patients in the adjuvant setting. Hum Vaccin Immunother.

[19] Eißler N, Mysliwietz J, Deppisch N, Ruf P, Lindhofer H, Mocikat R (2013). Potential of the trifunctional bispecific antibody surek depends on dendritic cells: rationale for a new approach of tumor immunotherapy. Mol Med.

[20] Seimetz D, Lindhofer H, Bokemeyer C (2010). Development and approval of the trifunctional antibody catumaxomab (anti-EpCAM x anti-CD3) as a targeted cancer immunotherapy. Cancer Treat Rev.

[21] Haraguchi M, Yamashiro S, Yamamoto A, Furukawa K, Takamiya K, Lloyd KO, Shiku H, Furukawa K (1994). Isolation of GD3 synthase gene by expression cloning of GM3 alpha-2,8-sialyltransferase cDNA using anti-GD2 monoclonal antibody. Proc Natl Acad Sci USA.

[22] Ragupathi G, Livingston PO, Hood C, Gathuru J, Krown SE, Chapman PB, Wolchok JD, Williams LJ, Oldfield RC, Hwu WJ (2003). Consistent antibody response against ganglioside GD2 induced in patients with melanoma by a GD2 lactone-keyhole limpet hemocyanin conjugate vaccine plus immunological adjuvant QS-21. Clin Cancer Res.

[23] Navid F, Santana VM, Barfield RC (2010). Anti-GD2 antibody therapy for GD2-expressing tumors. Curr Cancer Drug Targets.

[24] Ruf P, Jäger M, Ellwart J, Wosch S, Kusterer E, Lindhofer H (2004). Two new trifunctional antibodies for the therapy of human malignant melanoma. Int J Cancer.

[25] Ruf P, Schäfer B, Eißler N, Mocikat R, Hess J, Plöscher M, Wosch S, Suckstorff I, Zehetmeier C, Lindhofer H (2012). Ganglioside GD2-specific trifunctional surrogate antibody Surek demonstrates therapeutic activity in a mouse melanoma model. J Transl Med.

[26] Deppisch N, Ruf P, Eißler N, Neff F, Buhmann R, Lindhofer H, Mocikat R (2015). Efficacy and tolerability of a GD2-directed trifunctional bispecific antibody in a preclinical model: Subcutaneous administration is superior to intravenous delivery. Mol Cancer Ther.

[27] Egeter O, Mocikat R, Ghoreschi K, Dieckmann A, Röcken M (2000). Eradication of disseminated lymphomas with CpG-DNA-activated Th1 cells from non-transgenic mice. Cancer Res.

[28] Ziegler A, Heidenreich R, Braumüller H, Wolburg H, Weidemann S, Mocikat R, Röcken M (2009). EpCAM, a human tumor-associated antigen, promotes Th2 development and tumor immune evasion. Blood.

[29] Leach DR, Krummel MF, Allison JP (1996). Enhancement of antitumor immunity by CTLA-4 blockade. Science.

[30] Grosso JF, Jure-Kunkel MN (2013). CTLA-4 blockade in tumor models: An overview of preclinical and translational research. Cancer Immun.

[31] Morecki S, Lindhofer H, Yacovlev E, Gelfand Y, Slavin S (2006). Use of trifunctional bispecific antibodies to prevent graft versus host disease induced by allogeneic lymphocytes. Blood.

[32] Junttila TT, Li J, Johnston J, Hristopoulos M, Clark R, Ellerman D, Wang B-E, Li Y, Mathieu M, Li G, Young J, Luis E, G Lewis Phillips (2014). Antitumor efficacy of a bispecific antibody that targets HER2 and activates T cells. Cancer Res.

[33] Krupka C, Kufer P, Kischel R, Zugmaier G, Lichtenegger FS, Köhnke T, Vick B, Jeremias I, Metzeler KH, Altmann T, Schneider S, Fiegl M, Spiekermann K (2016). Blockade of the PD-1/PD-L1 axis augments lysis of AML cells by the CD33/CD3 BiTE antibody construct AMG 330: reversing a T-cell-induced immune escape mechanism. Leukemia.

[34] Lüking C, Kronenberger K, Frankenberger B, Nößner E, Röcken M, Mocikat R (2008). Antitumor effector functions of T cells are dependent on in vivo priming and restricted T-cell receptor expression. Int J Cancer.

[35] Kronenberger K, Nößner E, Frankenberger B, Wahl U, Dreyling M, Hallek M, Mocikat R (2008). A polyvalent cellular vaccine induces T-cell responses against specific self antigens overexpressed in chronic-lymphocytic B-cell leukemia. J Immunother.

[36] Schüler T, Qin Z, Ibe S, Noben-Trauth N, Blankenstein T (1999). T helper cell type 1-associated and cytotoxic T lymphocyte-mediated tumor immunity is impaired in interleukin-4-deficient mice. J Exp Med.

[37] Biedermann T, Zimmermann S, Himmelrich H, Gumy A, Egeter O, Sakrauski AK, Seegmüller I, Voigt H, Launois P, Levine AD, Wagner H, Heeg K, Louis JA (2001). IL-4 instructs TH1 responses and resistance to Leishmania major in susceptible BALB/c mice. Nat Immunol.

[38] Tang AL, Teijaro JR, Njau MN, Chandran SS, Azimzadeh A, Nadler SG, Rothstein DM, Farber DL (2008). CTLA4 expression is an indicator and regulator of steady-state CD4+ FoxP3+ T cell homeostasis. J Immunol.

[39] Goldstein MJ, Kohrt HE, Houot R, Varghese B, Lin JT, Swanson E, Levy R (2012). Adoptive cell therapy for lymphoma with CD4 T cells depleted of CD137-expressing regulatory T cells. Cancer Res.

[40] Wing JB, Ise W, Kurosaki T, Sakaguchi S (2014). Regulatory T cells control antigen-specific expansion of Tfh cell number and humoral immune responses via the coreceptor CTLA-4. Immunity.

